# Associations between Sclerostin and Anthropometric and Metabolic Parameters in Children and Adolescents

**DOI:** 10.3390/children8090788

**Published:** 2021-09-09

**Authors:** Shin-Hee Kim, Yun Jung Choi, Moon Bae Ahn, Won Kyoung Cho, Kyoung Soon Cho, Min Ho Jung, Byung-Kyu Suh

**Affiliations:** 1 Department of Medicine, Graduate School, Kyung Hee University, Seoul 02447, Korea; kshped@cathilic.ac.kr; 2 Department of Pediatrics, College of Medicine, The Catholic University of Korea, Seoul 06591, Korea; blue217@hanmail.net (Y.J.C.); mbahn@catholic.ac.kr (M.B.A.); wendy626@catholic.ac.kr (W.K.C.); soon926@catholic.ac.kr (K.S.C.); suhbk@catholic.ac.kr (B.-K.S.); 3 Department of Pediatrics, Yeouido St. Mary’s Hospital, College of Medicine, The Catholic University of Korea, Seoul 07345, Korea

**Keywords:** sclerostin, HOMA–IR, obesity

## Abstract

(1) Background: Bone plays an important role in the regulation of the systemic glucose and energy metabolism. Sclerostin, secreted by osteocytes, is an inhibitor of the Wnt/β–catenin bone metabolic pathway, and is involved in osteoporosis and metabolic disease. The aim of this study was to investigate the relationship between sclerostin and anthropometric and metabolic parameters in children and adolescents with obesity or who are overweight. (2) Methods: This study included 63 children and adolescents (20 obese, 11 overweight and 32 healthy control subjects). We evaluated the correlation between serum sclerostin and anthropometric parameters, metabolic parameters related to glucose (homeostasis model assessment of insulin resistance [HOMA–IR]), lipid, and bone metabolism (osteocalcin and 25-hydroxy vitamin D). (3) Results: Sclerostin and osteocalcin levels did not differ between obese and control groups. Sclerostin level was higher in boys than in girls (median 20.7 vs. 18.9 pmol/L, respectively; *p* = 0.04). In all subjects, sclerostin levels were negatively correlated with fasting insulin (r = −0.26; *p* = 0.04) and HOMA–IR (r = −0.28; *p* = 0.03), and positively correlated with serum concentrations of triglycerides (r = 0.29; *p* = 0.04), alkaline phosphatase (r = 0.41; *p* = 0.002), and osteocalcin (r = 0.33; *p* = 0.008). In obese patients, sclerostin levels were correlated negatively with fasting glucose (r = −0.49; *p* = 0.03) and HOMA–IR (r = −0.48; *p* = 0.03) and positively correlated with triglyceride levels (r = 0.53; *p* = 0.02). In the healthy control, sclerostin levels were correlated negatively with fasting insulin levels (r = −0.61; *p* < 0.001) and HOMA–IR (r = −0.36; *p* = 0.04). After adjusting for age, sex, and height SDS, a negative correlation between sclerostin and HOMA–IR was found (r = −0.39; *p* = 0.003) in all of the subjects. This association was more evident in obese patients (r = −0.60; *p* = 0.01) than in healthy controls (r = −0.39; *p* = 0.047). (4) Conclusions: Among children and adolescents with obesity, serum sclerostin was negatively correlated with HOMA–IR. Further studies are needed to clarify the mechanisms involved to understand how sclerostin affects the glucose metabolism.

## 1. Introduction

Previous studies have revealed a close relationship between cytokines produced by bone and energy metabolism, insulin resistance, and growth [1]. Osteocalcin is a bone protein mainly secreted by osteoblasts that has several extra-bone activities also related to the glucose metabolism [2].

Sclerostin, the product of the *SOST* gene, is a 213-amino acid glycoprotein mainly secreted by osteocytes [3]. Inactivating mutations in *SOST* are associated with significant increases in rates of bone formation and bone mass [4]. Meanwhile, mice lacking a functional *SOST* gene showed a marked reduction in fat mass with enhanced glucose tolerance and insulin sensitivity [5]. Sclerostin is considered to be a local inhibitor of the skeletal *Wnt* signaling pathway, which has a direct role in osteoblast differentiation, proliferation, and activity [6]. In addition, the Wnt signaling pathway plays an important role in the pathogenesis of metabolic disease by involving in pancreatic β-cell proliferation, lipid metabolism, and glucose-induced insulin secretion [7,8].

In an adult study, serum sclerostin level increased in patients with type 2 diabetes compared to healthy controls and showed a positive correlation with body mass index (BMI) and fat mass [9]. Other studies have reported a positive correlation between sclerostin levels and the homeostasis model assessment of insulin resistance (HOMA–IR) in prediabetic adults [1] and those with type 2 diabetes [10]. A few studies have investigated the relationship between sclerostin and metabolic parameters in children and adolescents, with conflicting results [11,12].

We aimed to evaluate the associations between serum sclerostin and anthropometric and metabolic parameters in children and adolescents with obesity.

## 2. Patients and Methods

### 2.1. Participants

Sixty-three Korean children and adolescents (32 males and 31 female) who underwent a health examination at the Yeouido St. Mary’s Hospital between March 2013 and February 2014 were included in this study. Of the participants, 20 (31.7%) were obese, 11 (17.5%) were overweight, and 32 (50.8%) were healthy controls. The median age for obese, overweight, and healthy control groups were 10.9, 10.2, and 10.3 years, respectively. Of the 63 subjects, 24 (38.1%) were Tanner stage 1, 29% (46.0%) were Tanner stage 2–3, and 10 (15.9%) were Tanner stage 4–5. Exclusion criteria included subjects with chronic diseases (involving respiratory, gastrointestinal, and cardiovascular systems), genetic disorders (including Turner syndrome, Prader-Willi syndrome and congenital adrenal hypoplasia), and endocrine diseases associated with obesity or being overweight. This study was approved by the local Institutional Review Board (IRB number: SC12TISI0032) with informed consent obtained from all participants.

### 2.2. Anthropometry Measurements

All patients included underwent physical examinations, and standard deviation scores (SDSs) of weight, height, and BMI were calculated according to the 2007 Korean National Growth chart [13]. Subjects with BMI ≥ 95th, 85th–94th, and 5th–84th percentile for age and sex were considered overweight, obese, and normal weight, respectively [13]. Bone age was determined by comparing the left wrist radiographs of the subject with the nearest matching reference radiographs displayed in the atlas of Greulich and Pyle [14]. Pubertal status was determined following Marshall and Tanner [15]. Onset of puberty was defined as a testicular volume ≥ 4 mL or Tanner breast stage ≥ B2.

### 2.3. Laboratory Evaluations

Venous blood samples were obtained in the morning after 10 h of overnight fasting. Samples were assayed for glucose, insulin, insulin-like growth factor 1 (IGF-1), lipid profile, thyroid function tests, alkaline phosphatase (ALP), 25-hydroxy vitamin D, osteocalcin, and sclerostin levels. Serum glucose, total cholesterol (TC), low-density lipoprotein cholesterol (LDL-C), high-density lipoprotein cholesterol (HDL-C), triglycerides (TG), and ALP were tested using a Beckman Coulter AU5800 clinical chemistry analyzer and the manufacturer’s reagents (Beckman Coulter, Brea, CA, USA). Serum insulin levels were measured with Elecsys insulin Assay (Cobas e411 immunoassay analyzer; Roche Diagnostics, Mannheim, Germany). IGF-1 was measured by an enzyme-labeled chemiluminescent immunometric assay (Immulite 2000; Siemens Medical Solutions Diagnostics, Los Angeles, CA, USA). HbA1c levels were measured using automated high-performance liquid chromatography (HLC-723 G7, Tosoh, Tokyo, Japan). Serum 25-hydroxy vitamin D was measured with a Siemens ADVIA Centaur^®^ vitamin D TOTAL immunoassay (Siemens Healthcare, Erlangen, Germany). Serum osteocalcin was measured using an electrochemiluminescence immunoassay (ECLIA) and Elecsys autoanalyzer (Roche Diagnostics GmbH, Mannheim, Germany). Serum sclerostin levels were measured by enzyme-linked immunosorbent assay (Biomedica, Vienna, Austria, detection limit: 7 pmol/L). The intra- and inter-assay coefficients of variation for serum sclerostin were ≤7% and ≤10%, respectively.

HOMA–IR was calculated with following formula: fasting insulin (μU/mL) × fasting glucose (mmol/L)/22.5. Dyslipidemia was defined as TC ≥ 200 mg/dL, LDL-C ≥ 130 mg/dL, HDL-C ≤ 35 mg/dL, and TG ≥ 150 mg/dL, or a combination thereof [16,17].

### 2.4. Statistical Analysis

All statistical analyses were performed in SPSS for Windows version 18 (SPSS Inc., Chicago, IL, USA). The significance of differences in proportions between groups was tested using the chi-squared test or Fisher’s exact test, as appropriate. The significance of differences in continuous variables between groups was assessed by the Mann-Whitney U-test or Kruskal–Wallis test. Post-hoc analyses were conducted with Bonferroni correction for multiple comparisons, and post-hoc adjusted *p* values were reported. The correlation of sclerostin level with age, anthropometric parameters (height SDS and BMI SDS), metabolic parameters related to glucose (insulin, IGR-1, and HOMA–IR), lipid, and bone metabolism (osteocalcin and 25-hydroxy vitamin D) were examined using Spearman’s correlation, Spearman’s partial correlation, and multiple linear regression. All *p* values < 0.05 were considered statistically significant.

## 3. Results

### 3.1. Clinical Characteristics and Laboratory Data of the Participants

The clinical characteristics of the obese, overweight, and healthy subjects are shown in Table 1. There were no differences in age or the distribution of sex and pubertal development between the three groups. There were significant differences in height SDS, BMI, BMI SDS, fasting insulin, HOMA–IR, and total cholesterol between three groups. Post-hoc analysis using Bonferroni correction indicated that patients with obesity had higher values of height SDS, BMI, BMI SDS, HOMA–IR, and total cholesterol than healthy control subjects. Patients with obesity were more likely to have dyslipidemia than control subjects (60.0% vs. 14.3%, respectively; adjusted *p* = 0.007). There were no significant differences between obese and control subjects in osteocalcin levels (median 59.1 vs. 78.6 μg/L, respectively; adjusted *p* = 0.11) and sclerostin levels (median 19.6 vs. 20.2 pmol/L, respectively; adjusted *p* > 0.99). Sclerostin levels were higher in boys than in girls (median 20.7 vs. 18.9 pmol/L, respectively; *p* = 0.04). Sclerostin levels were not different between prepubertal and pubertal subjects (median 20.5 vs. 19.6 pmol/L, respectively; *p* = 0.17). Among 39 pubertal subjects, sclerostin levels were not different between early (Tanner stage 2–3) and late (Tanner stage 4–5) pubertal subjects (median 20.9 vs. 19.2 pmol/L, respectively; *p* = 0.55). Demographic, clinical, and laboratory data for prepubertal, early pubertal, and late pubertal subjects are shown in Table S1.

### 3.2. Associations between Serum Sclerostin Level and Anthropometric Parameters

Associations between serum sclerostin levels and anthropometric parameters were analyzed (Table 2). In all subjects, chronological age, bone age, height SDS and BMI SDS were not associated with serum sclerostin level. In obese subjects, height SDS was positively associated with sclerostin level.

### 3.3. Association between Serum Sclerostin Level and Metabolic Parameters

**Figure 1:**
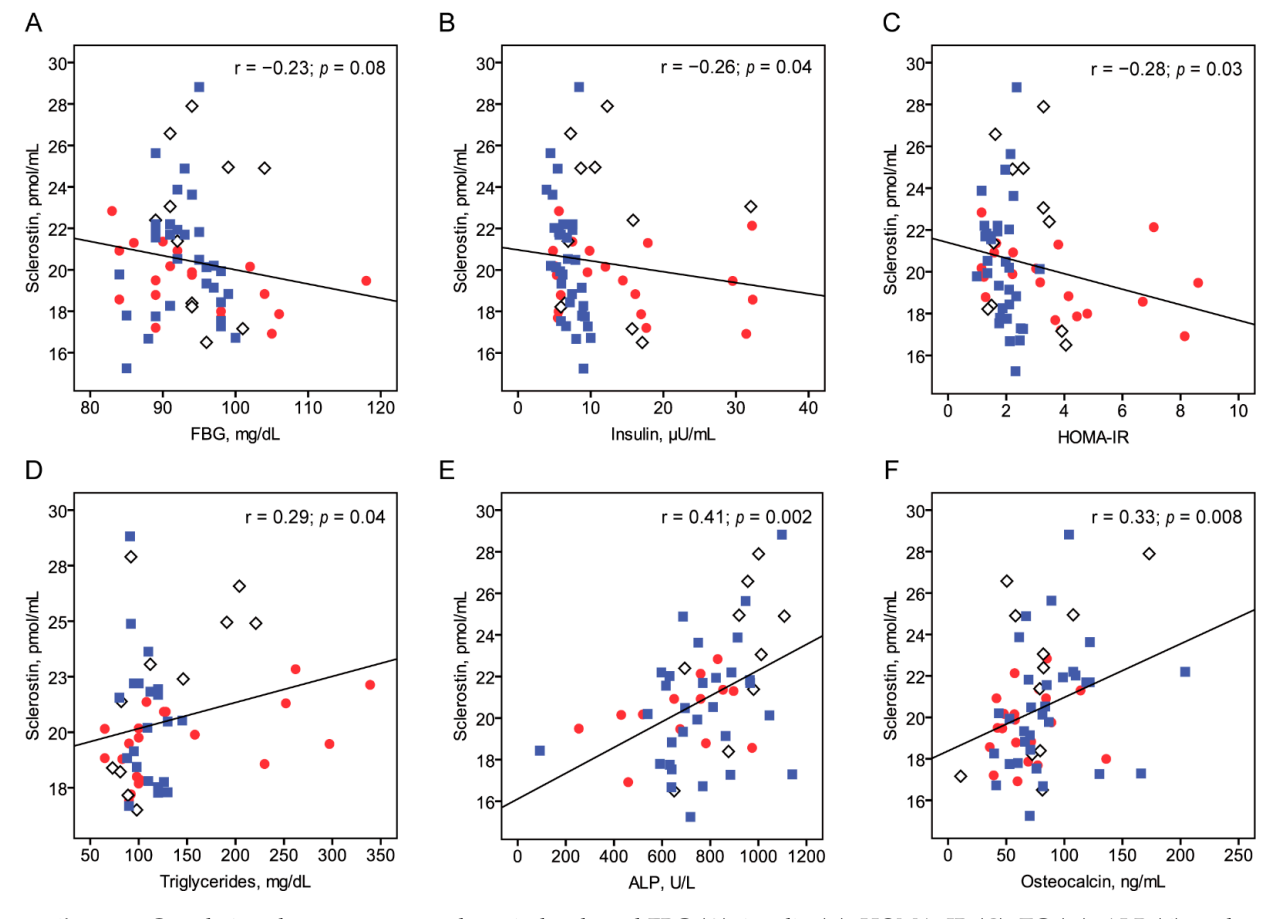
Correlations between serum sclerostin levels and FBG (**A**), insulin (**B**), HOMA–IR (**C**), TG (**D**), ALP (**E**), and osteocalcin (**F**). FBG, fasting blood glucose; HOMA–IR, homeostasis model assessment–insulin resistance; ALP, alkaline phosphatase. Red closed circles, open diamonds, and blue closed rectangles indicate the data for obese, overweight, and control groups, respectively.

Associations between serum sclerostin levels and metabolic parameters were analyzed (Table 3, Figure 1). Among all subjects, sclerostin level correlated negatively with fasting insulin level (r = −0.26; *p* = 0.04) and HOMA–IR (r = −0.28; *p* = 0.03) and was positively correlated with triglyceride level (r = 0.29; *p* = 0.04), ALP (r = 0.41; *p* = 0.002) and osteocalcin (r = 0.33; *p* = 0.008). After controlling for age, sex, and height SDS, sclerostin level was correlated negatively with fasting insulin level (r = −0.38; *p* = 0.003) and HOMA–IR (r = −0.39; *p* = 0.003) and positively correlated with ALP (r = 0.36; *p* = 0.01) and osteocalcin (r = 0.36; *p* = 0.006) (Table 4). Multiple linear regression analysis indicated that HOMA–IR (β = –0.34; *p* = 0.02), triglycerides (β = 0.41; *p* = 0.004), and osteocalcin (β = 0.26; *p* = 0.049) were independently correlated with sclerostin level.

In the group of obese patients, sclerostin level was correlated negatively with fasting glucose (r = −0.49; *p* = 0.03) and HOMA–IR (r = −0.48; *p* = 0.03) and positively correlated with triglyceride level (r = 0.53; *p* = 0.02). After controlling for age, sex, and height SDS, sclerostin levels correlated negatively with fasting glucose levels (r = −0.50; *p* = 0.04) and HOMA–IR (r = −0.60; *p* = 0.01) (Table 4).

In the healthy control group, sclerostin level correlated negatively with fasting insulin level (r = −0.61; *p* < 0.001) and HOMA–IR (r = −0.36; *p* = 0.04). After controlling for age, sex, and height SDS, sclerostin levels correlated negatively with fasting insulin levels (r = −0.67; *p* < 0.001) and HOMA–IR (r = −0.39; *p* = 0.047) (Table 4).

## 4. Discussion

The key finding of this study is that serum sclerostin was negatively correlated with HOMA–IR in children and adolescents. This association was more evident among obese patients.

There is limited literature evaluating the association between serum sclerostin and parameters related to glucose metabolism in children and adolescents. Our study showed that serum sclerostin was negative correlated with HOMA-–IR but not correlated with insulin in obese patients. A previous study of children and adolescents with obesity found no association between serum sclerostin and fasting insulin or HOMA-–IR [11]. Another study of children and adolescents with obesity reported a negative correlation of sclerostin level with both fasting insulin and HOMA-–IR [12]. The reasons for different correlations being found between fasting insulin or HOMA-–IR and serum sclerostin may be related to differences in study population and/or issues with the validity of surrogate markers for insulin resistance in children and adolescents. The difference in magnitude of obesity and glucose intolerance between study populations may contribute to discrepant results among different studies. Another possible reason for divergent results may be the difference in purbertal stage of the subjects in various studies. Children normally experience transient insulin resistance during puberty [18]. Moran et al. evaluated 357 normal children who underwent euglycemic clamp studies and showed that insulin resistance increased at Tanner stage 2 and reached a peak at Tanner stage 3, but returned to near prepubertal levels by Tanner stage 5 [18]. Fasting insulin is not always an optimal tool for the assessment of insulin resistance in pediatric patients [19,20]. Measurement of fasting insulin as a surrogate for insulin resistance is also limited by the multitude of different assays used for the determination of insulin. These different assays can show up to a twofold variation in insulin concentrations [21]. HOMA–IR is a more accurate tool for the assessment of insulin sensitivity than fasting plasma insulin since it assesses the relationship between the functioning of β-cells and insulin resistance [22]. Despite this, some authors argue that measuring HOMA does not offer any advantages over measuring fasting insulin in euglycemic children [23]. Further studies including a large number of cases with more reliable analysis methods, such as using a hyperinsulinemic euglycemic clamp, are required to clarify this issue.

We observed no correlations between sclerostin levels and TC, LDL-C, HDL-C, or TG levels. In addition, sclerostin levels were not different between subjects with or without dyslipidemia (data not shown). Previous studies reported an association between Wnt/β-catenin signaling and hyperlipidemia [24]. *SOST* knock-out mice have been shown to have significantly less body fat and smaller adipocytes than wild mice. Inversely, mice with overexpression of *SOST* present with excess adipose tissue [5,25].

In this study, serum sclerostin levels were significantly higher in boys than girls, as has been observed in adults. The circulating sclerostin levels might reflect total body skeletal mass, which would explain the higher levels in males due to their larger skeleton [26]. We found that sclerostin levels were not different between prepubertal and pubertal subjects. Kirmani et al. showed that the increase in sex steroids during puberty may decrease sclerostin levels later in puberty [27]. Previous studies have reported an inverse association between serum sclerostin and estrogen levels in adolescent girls [27], and have shown that estrogen reduces circulating sclerostin levels in adults [26]. However, this phenomenon is difficult to explain based on sex steroid levels, as estradiol levels increase from early puberty and late puberty to adulthood. Another study showed that sex steroid levels increased and IGF-1 levels began to decrease after puberty [28]; this IGF-1 decrease might be related to the increase in sclerostin level observed in adults.

We a positive correlation between sclerostin and osteocalcin in all subjects, but we found this association was especially strong in obese patients. Previous studies reported a positive correlation between sclerostin and osteocalcin levels in children with type 1 diabetes [29] and in obese children and adolescents [12]. Osteocalcin, a marker of bone formation, acts as a hormone by stimulating insulin production and increasing energy expenditure and insulin sensitivity in target organs [30]. Sclerostin affects the activity of osteoblasts on the bone surface as a paracrine-acting regulator [31]. This could explain the correlation between sclerostin and osteocalcin levels. Both osteocalcin and sclerostin play a central role in glucose metabolism through different pathways [32]. Further research is needed to determine the interplay between sclerostin and osteocalcin in the insulin–glucose metabolism.

In our study, there were no differences in sclerostin levels between children and adolescents with obesity and normal-weight subjects. Likewise, other recent studies have not observed differences in sclerostin levels between obese and non-obese children [33,34]. However, other researchers have reported a positive relationship between sclerostin levels and BMI SDS in children and adolescents [11]. Despite these conflicting findings, sclerostin has been shown to be associated with several inflammatory and metabolic conditions. In children, sclerostin levels have been found to correlate negatively with leptin levels and correlate positively with adiponectin levels [29]. These data suggest that this protein is not only a regulator of bone mass, but also of the energy metabolism.

This study had several limitations. First, our small sample size limited our ability to accurately determine the strength of associations or trends. Second, furthermore, the cross-sectional nature of this study limited our ability to infer causality. The effect of certain possible confounding factors, such as physical activity and dietary factors, were not investigated in this study. Previous studies showed that serum sclerostin level was negatively correlated with physical activity in healthy adults [35], whereas a positive correlation was reported in adolescent females [36]. Diet-induced weight loss in type 2 diabetes was reported to induce significant increases in born turnover and serum sclerostin levels [37]. Further matched case-control studies and longitudinal studies with a larger number of patients are required to clarify this issue.

In conclusion, we found that serum sclerostin level was negatively correlated with HOMA–IR in children and adolescents with obesity. Further studies are needed to clarify the mechanisms that determine how sclerostin affects glucose metabolism.

## Figures and Tables

**Table 1:** Demographic, clinical, and laboratory data for obese, overweight, and control participants.

Characteristic	All Subjects (*n* = 63)	Obese (*n* = 20)	Overweight (*n* = 11)	Control (*n* = 32)	*p* Value	
					All	Obese vs. Control ^a^
Age, years	10.9 (6.4–17.5)	10.2 (6.4–17.5)	10.3 (7.7–16.1)	11.2 (8.1–14.4)	0.90	>0.99
Male sex	32/63 (50.8)	13/20 (65.0)	5/11 (45.5)	14/32 (43.8)	0.31 ^b^	0.41 ^b^
Height SDS	0.57 (−1.28–2.85)	1.06 (−0.83–2.85)	1.00 (−0.05–2.25)	−0.56 (−1.28–2.34)	<0.001	0.001
BMI, kg/m^2^	21.0 (13.9–36.0)	26.8 (21.5–36.0)	21.6 (18.7–25.5)	17.9 (13.9–21.9)	<0.001	<0.001
BMI SDS	1.02 (−1.91–3.03)	2.16 (1.70–3.03)	1.37 (1.02–1.63)	0.06 (−1.91–0.93)	<0.001	<0.001
Puberty	39/63 (61.9)	10/20 (50.0)	8/11 (72.7)	21/32 (65.6)	0.38 ^b^	0.79 ^b^
Fasting glucose, mg/dL	93 (83–118)	92 (83–118)	94 (89–104)	93 (84–100)	0.60	>0.99
Fasting insulin, μU/mL	7.5 (3.9–32.3)	10.9 (4.8–32.3)	10.6 (5.9–32.0)	6.7 (3.9–10.0)	0.005	0.051
IGF-1, ng/mL	286 (85–758)	209 (85–539)	329 (195–758)	279 (137–652)	0.39	>0.99
HOMA–IR	2.1 (1.0–8.6)	3.4 (1.1–8.6)	2.6 (1.4–4.1)	2.0 (1.0–3.2)	0.01	0.03
Total cholesterol, mg/dL ^b^	180 (120–240)	190 (151–240)	169 (122–198)	168 (120–185)	<0.001	<0.001
LDL cholesterol, mg/dL ^c^	105 (71–198)	125 (76–198)	91 (71–173)	96 (88–135)	0.002	0.01
HDL cholesterol, mg/dL ^c^	50 (32–95)	47 (36–89)	45 (32–64)	51 (35–95)	0.28	0.49
Triglycerides, mg/dL ^c^	105 (65–339)	101 (65–339)	98 (73–221)	110 (80–145)	0.82	>0.99
Dyslipidemia ^c^	20/52 (38.5)	12/20 (60.0)	5/11 (45.5)	3/21 (14.3)	0.009 ^b^	0.007 ^b^
ALP, U/L	765 (92–1141)	760 (254–974)	956 (650–1108)	748 (92–1141)	0.03	>0.99
25-hydroxy vitamin D, ng/mL	13.1 (5.2–25.1)	11.9 (5.9–16.1)	10.8 (5.2–16.7)	13.8 (6.0–25.1)	0.08	0.24
Osteocalcin, ng/mL	72.4 (10.8–204.0)	59.1 (35.8–136.0)	79.2 (10.8–173.0)	78.6 (39.5–204.0)	0.10	0.11
Sclerostin, pmol/L	20.1 (15.2–28.8)	19.6 (16.9–22.8)	22.4 (16.5–27.9)	20.2 (15.2–28.8)	0.25	>0.99

Data are median values (range) for continuous variables and number of cases (%) for categorical variables, unless otherwise specified. SDS, standard deviation score; BMI, body mass index; IGF-1, insulin-like growth factor-1; HOMA–IR, homeostasis model assessment-insulin resistance; LDL, low-density lipoprotein; HDL, high-density lipoprotein; ALP, alkaline phosphatase. ^a^ Bonferroni-adjusted *p* values. ^b^ *p* values represent the statistical significance of the differences in the proportion of the patients with male sex, puberty, and dyslipidemia between groups. ^c^ Measured in 52 participants: 20 obese, 11 overweight, and 21 control subjects.

**Table 2:** Correlation between serum sclerostin and anthropometric parameters.

Variable	All Subjects (*n* = 63)		Obese (*n* = 20)		Overweight (*n* = 11)		Control (*n* = 32)	
	r	*p* Value	r	*p* Value	r	*p* Value	r	*p* Value
Chronological age, years	0.11	0.40	−0.002	0.99	0.26	0.45	0.22	0.23
Bone age, years	0.25	0.06	0.20	0.43	0.95	<0.001	0.21	0.26
Height SDS	0.22	0.09	0.50	0.02	0.34	0.31	0.05	0.80
BMI SDS	−0.001	>0.99	0.29	0.21	−0.26	0.43	0.06	0.74

SDS, standard deviation score; BMI, body mass index.

**Table 3:** Correlation between serum sclerostin level and metabolic parameters.

Variable	All Subjects (*n* = 63)		Obese (*n* = 20)		Overweight (*n* = 11)		Control (*n* = 32)	
	r	*p* Value	r	*p* Value	r	*p* Value	r	*p* Value
HbA_1C_	0.05	0.79	0.18	0.45	0.16	0.64	−0.46	0.35
Fasting glucose, mg/dL	−0.23	0.08	−0.49	0.03	−0.21	0.54	−0.20	0.28
Fasting insulin, μU/mL	−0.26	0.04	−0.21	0.38	−0.02	0.96	−0.61	<0.001
IGF-1, ng/mL	0.15	0.36	−0.32	0.48	0.43	0.24	0.14	0.54
HOMA–IR	−0.28	0.03	−0.48	0.03	−0.13	0.71	−0.36	0.04
Total cholesterol, mg/dL ^a^	−0.10	0.50	0.31	0.19	−0.40	0.23	−0.15	0.51
LDL cholesterol, mg/dL ^a^	−0.01	0.92	0.30	0.19	−0.14	0.69	0.17	0.48
HDL cholesterol, mg/dL ^a^	−0.15	0.28	−0.002	0.99	−0.39	0.23	−0.37	0.10
Triglycerides, mg/dL ^a^	0.29	0.04	0.53	0.02	0.58	0.06	−0.24	0.30
ALP, U/L	0.41	0.002	0.30	0.31	0.55	0.13	0.26	0.16
Osteocalcin, ng/mL	0.33	0.008	0.19	0.43	0.36	0.29	0.34	0.06
25-hydroxy vitamin D, ng/mL	−0.04	0.76	−0.32	0.17	0.13	0.73	−0.02	0.91

IGF-1, insulin-like growth factor-1; HOMA–IR, homeostasis model assessment–insulin resistance; LDL, low-density lipoprotein; HDL, high-density lipoprotein; ALP, alkaline phosphatase. ^a^ Measured in 52 participants: 20 obese, 11 overweight, and 21 control subjects.

**Table 4:** Partial correlation between serum sclerostin level and metabolic parameters ^a^.

Variable	All Subjects (*n* = 63)		Obese (*n* = 20)		Control (*n* = 32)	
	r	*p* Value	r	*p* Value	r	*p* Value
HbA_1C_	−0.05	0.76	0.01	0.97	−0.53	0.36
Fasting glucose, mg/dL	−0.23	0.09	−0.50	0.04	−0.22	0.28
Fasting insulin, μU/mL	−0.38	0.003	−0.38	0.13	−0.67	<0.001
IGF-1, ng/mL	0.12	0.51	−0.19	0.81	0.03	0.90
HOMA–IR	−0.39	0.003	−0.60	0.01	−0.39	0.047
Total cholesterol, mg/dL ^b^	−0.19	0.20	0.28	0.28	−0.05	0.85
LDL cholesterol, mg/dL ^b^	−0.12	0.41	0.32	0.21	0.05	0.84
HDL cholesterol, mg/dL ^b^	−0.12	0.42	−0.08	0.76	−0.39	0.12
Triglycerides, mg/dL ^b^	0.22	0.14	0.35	0.17	−0.33	0.19
ALP, U/L	0.36	0.01	0.03	0.94	0.24	0.25
Osteocalcin, ng/mL	0.36	0.006	0.14	0.60	0.31	0.12
25-hydroxy vitamin D, ng/mL	0.11	0.42	−0.13	0.61	0.06	0.77

IGF-1, insulin-like growth factor-1; HOMA–IR, homeostasis model assessment–insulin resistance; LDL, low-density lipoprotein; HDL, high-density lipoprotein; ALP, alkaline phosphatase. ^a^ Controlled for age, sex, and height SDS. ^b^ Measured in 52 participants: 20 obese, 11 overweight, and 21 control subjects.

## Data Availability

The raw data supporting the conclusions of this article will be made available by the authors without undue reservation.

## Supplementary Materials

The following are available online at https://www.mdpi.com/article/10.3390/children8090788/s1. Table S1: Demographic, clinical, and laboratory data for pre-, early, and late pubertal subjects.
