# Supplementary material for: Associations between Sclerostin and Anthropometric and Metabolic Parameters in Children and Adolescents
Source: Children (Basel). 2021 Sep 9;8(9):788. doi: 10.3390/children8090788 (PMC8467097; doi:10.3390/children8090788)
Supplement: Supplementary file 1 [file children-08-00788-s001.zip › children-1303111-supplementary.pdf]

Table S1. Demographic, clinical, and laboratory data for pre-, early, and late pubertal subjects

Characteristic	Prepubertal subjects (Tanner stage 1) (n = 24)	Early pubertal subjects (Tanner stage 2–3) (n = 29)	Late pubertal subjects (Tanner stage 4–5) (n = 10)	*p* value	
				Overall[a]	Early vs. late pubertal subjects
Age, years	9.2 (8.5–10.4)	11.2 (9.5–12.2)	14.2 (12.9–15.2)	<0.001	<0.001
Male sex	16/32 (66.7)	11/29 (37.9)	5/10 (50.0)	0.11	0.71
Height SDS	0.81 (-0.28–2.85)	0.57 (-1.27–2.25)	0.07 (-0.93–2.34)	0.72	0.99
BMI, kg/m^2	20.6 (13.9–36.0)	21.0 (14.2–33.5)	25.3 (17.3–35.5)	0.23	0.12
BMI SDS	1.38 (-0.54–2.78)	0.64 (-1.91–3.03)	1.36 (-1.18–2.73)	0.83	0.72
Fasting glucose, mg/dL	94 (83–118)	92 (84–106)	92 (86–101)	0.64	0.72
Fasting insulin, mg/dL	7.7 (4.5–32.3)	6.9 (3.9–32.2)	9.9 (5.4–17.9)	0.29	0.11
IGF-1, ng/mL	204.2 (84.6–326.9)	328.9 (175.6–651.6)	490.5 (230.4–757.9)	<0.001	0.11
HOMA-IR	2.1 (1.2–8.6)	2.1 (1.0–7.1)	2.9 (1.7–4.8)	0.11	0.04
Total cholesterol, mg/dL[b]	187 (120–220)	170 (122–240)	174 (120–198)	0.04	0.90
LDL cholesterol, mg/dL[b]	110 (75–198)	95 (71–173)	105 (84–120)	0.37	0.94
HDL cholesterol, mg/dL[b]	53 (38–89)	50 (32–95)	44 (36–50)	0.047	0.07
Triglycerides, mg/dL[b]	109 (65–297)	110 (80–339)	95 (88–252)	0.73	0.31
Dyslipidemia[b]	7/16 (43.8)	11/26 (42.3)	2/10 (20.0)	0.41	0.27
ALP, U/L	758 (92–1108)	812 (520–1141)	640 (254–920)	0.22	0.09
25-hydroxy vitamin D, ng/mL	12.5 (7.0–25.1)	13.5 (6.0–23.4)	9.3 (5.2–16.7)	0.20	0.08
Osteocalcin, ng/mL	62.7 (35.8–118.9)	81.9 (43.6–173.0)	66.6 (10.8–204.0)	0.03	0.37
Sclerostin, pmol/L	19.7 (16.7–25.6)	20.9 (15.2–28.8)	19.2 (17.6–22.9)	0.54	0.55

Data are median values (range) for continuous variables and number of cases (%) for categorical variables, unless otherwise specified. SDS, standard deviation score; BMI, body mass index; IGF-1, insulin-like growth factor-1; HOMA-IR, homeostasis model assessment-insulin resistance; LDL, low-density lipoprotein; HDL, high-density lipoprotein; ALP, alkaline phosphatase. [a]The significance of differences in continuous variables between three groups was assessed by Kruskal-Wallis test.
[b]Measured in 52 participants: 16 prepubertal, 26 early pubertal and 10 late pubertal subjects.
